# The Complex Relation of Branched‐Chain Amino Acids and Inflammation in the Obesity and Diabetes Context

**DOI:** 10.1111/obr.70092

**Published:** 2026-01-22

**Authors:** Bernardo Starling‐Soares, Monique Macedo Coelho, Bruna Guerra Campolina, Camila Kümmel Duarte, Tatiani Uceli Maioli

**Affiliations:** ^1^ Programa de Pós‐Graduação em Bioquímica e Imunologia, Instituto de Ciências Biológicas Universidade Federal de Minas Gerais Belo Horizonte Brazil; ^2^ Departamento de Nutrição, Escola de Enfermagem Universidade Federal de Minas Gerais Belo Horizonte Brazil

**Keywords:** BCAA, biomarker, branched‐chain amino acid, diabetes, inflammation, obesity

## Abstract

In a scenario with increasing cases of obesity and diabetes worldwide, branched‐chain amino acids (BCAA) metabolism has become an important factor in the understanding of these pathologies. More recently, its chronic high plasma levels have been postulated, alongside glucose, inflammatory factors, and other molecules, as an important predictive marker for developing insulin resistance. High‐fat diet protocols and models mimicking obesity and type‐2 diabetes have clarified our knowledge about how these conditions, which have an important inflammatory aspect, impact the BCAA catabolism in several tissues and its systemic effects. On the other hand, BCAA supplementation has been studied in several experimental models aiming to understand its role in inflammation. Evidence reveals that a chronic low‐grade inflammatory state is an important factor in several age‐related pathological conditions and that its presence, characterized by augmented proinflammatory cytokines, high glucose and BCAA levels, would be a determining factor. Although, the relationship between BCAA and inflammation is complex and our current knowledge cannot identify a causative role for these amino acids, as in the majority of the cases a previous or concomitant stimulus was necessary to demonstrate their role in the modulation of inflammation.

**Abbreviations**
AA: amino acid
ApoE: apoliprotein E
ATM: adipose tissue macrophage
BAT: brown adipose tissue
BCAA: branched‐chain amino acid
BCAT: branched‐chain aminotransferase
BCKA: branched‐chain keto acid
BCKDH: branched‐chain ketoacid dehydrogenase
BCKDK: BCKDH kinase
BMDM: bone marrow derived macrophage
CD40L: Cluster Of Differentiation 40 Ligand
DBT: dihydrolipoamide branched chain transacylase E2
DMEM: Dulbecco's modified Eagle medium
eWAT: eppididimal white adipose tissue
HFD: high‐fat diet
HG: high glucose
IA: intrinsic activity
ICAM‐1: Intercellular Adhesion Molecule‐1
I: isoleucine
iNOS: Inducible Nitric Oxide Synthase
IRS: insulin receptor substrate
iWAT: inguinal white adipose tissue
L: leucine
LDL: low density lipoprotein
LPS: lipopolysacharides
LT: liver tissue
M0: macrophage
M1: classic‐activated macrophages
mTORC1: mammalian target of rapamycin complex
ND: normal diet
NF‐κB: transcription factor nuclear factor kappa B
NG: normal glucose
NO: oxide nitric
PBMC: peripheral blood mononuclear cells
PGE2: prostaglandin E2
PP2cm: BCKDH phosphatase
PT: pancreas tissue
RPMI: Roswell Park Memorial Institute medium
ROS: reactive oxygen species
SMT: skeletal muscle tissue
SOCS1: suppressors of cytokines signaling 1
SOCS3: suppressors of cytokines signaling 3
STZ: streptozotocin
T2D: type‐2 diabetes mellitus
V: valine
w: weeks
wo: weeks‐old
WT: wild‐type mice

## 1 Introduction

Chronic elevation levels of branched‐chain amino acids (BCAAs: leucine, isoleucine, and valine) in animal fluids have been recently incorporated into the list of predictive biomarkers, alongside glucose, inflammatory factors, and other molecules, for assessing the risk of developing insulin resistance and subsequently type‐2 diabetes mellitus (T2D) [1, 2, 3, 4, 5, 6, 7]. However, the discovery and study of these amino acids **(AAs)** in nature are described as early as the ninth century, with leucine being the first of all **AA** completely (free nature form and profiled from protein hydrolysis) identified [8, 9].

BCAAs are considered the most hydrophobic aliphatic **AA**s, as a consequence of the nonpolar branch derived from their skeleton carbon [10]. Thereby, fundamentally three BCAAs are found in nature as this number provides the minimum diversity necessary in aliphatic hydrophobic side chains for the structure and functioning of all proteins [10]. Thus, BCAA is found in high concentrations in nonaqueous environments, inside globular proteins, in transmembrane domains of proteins, and as a primary sequence of insoluble proteins [11]. From BCAA‐rich sequences in proteins, isoleucine, and valine take more part in β‐sheets, whereas leucine is found in α‐helices, especially in coiled‐coil α‐helices present in cytoskeleton proteins and motor proteins, from where it was initially obtained by Braconnot [9, 12].

BCAAs represent up to 35% of essential AA**s** content in some tissues and their metabolism presents a crucial role in energetic homeostasis and cellular nitrogen equilibrium [13, 14, 15, 16]. The transamination reaction of BCAA is a mechanism of nitrogen transfer from these AAs, depending on the tissue momentarily requirement, which can produce glutamate and consequently other nonessential AAs [17, 18]. It should be noted that mammalian branched‐chain aminotransferase (BCAT) is the first step of the catabolic BCAA pathway and is very specific for BCAA and glutamate, with substrate affinities as follows: leucine, isoleucine > valine > glutamate [19]. The second BCAA catabolism enzyme is the branched‐chain ketoacid dehydrogenase (BCKDH) complex, which is the main regulatory step in the pathway [20]. As the rate‐limiting enzyme, the activity of the BCKDH complex, unlike BCAT, is tightly modulated by a phosphorylation/dephosphorylation regulation [21, 22]. The enzyme BCKDH kinase (BCKDK) promotes the inactivation of BCKDH by phosphorylation of the E1a subunit, while the BCKDH phosphatase (PP2cm also called PPM1K) is responsible for the activation of the complex by dephosphorylation of the E1a subunit [21]. Activation of the BCKDH complex in the short term can be achieved by the modulatory inhibition of BCKDK enzyme activity by α‐KIC (alpha ketoisocaproate), which results from transamination of leucine [22]. On the other hand, the long‐term control mechanisms for catabolic activity in animal models include (i) a reduction or an increase in the gene expression of BCKDH **and** (ii) a decrease or increase in the expression of BCKDK and/or PP2cm [23].

**FIGURE 1:**
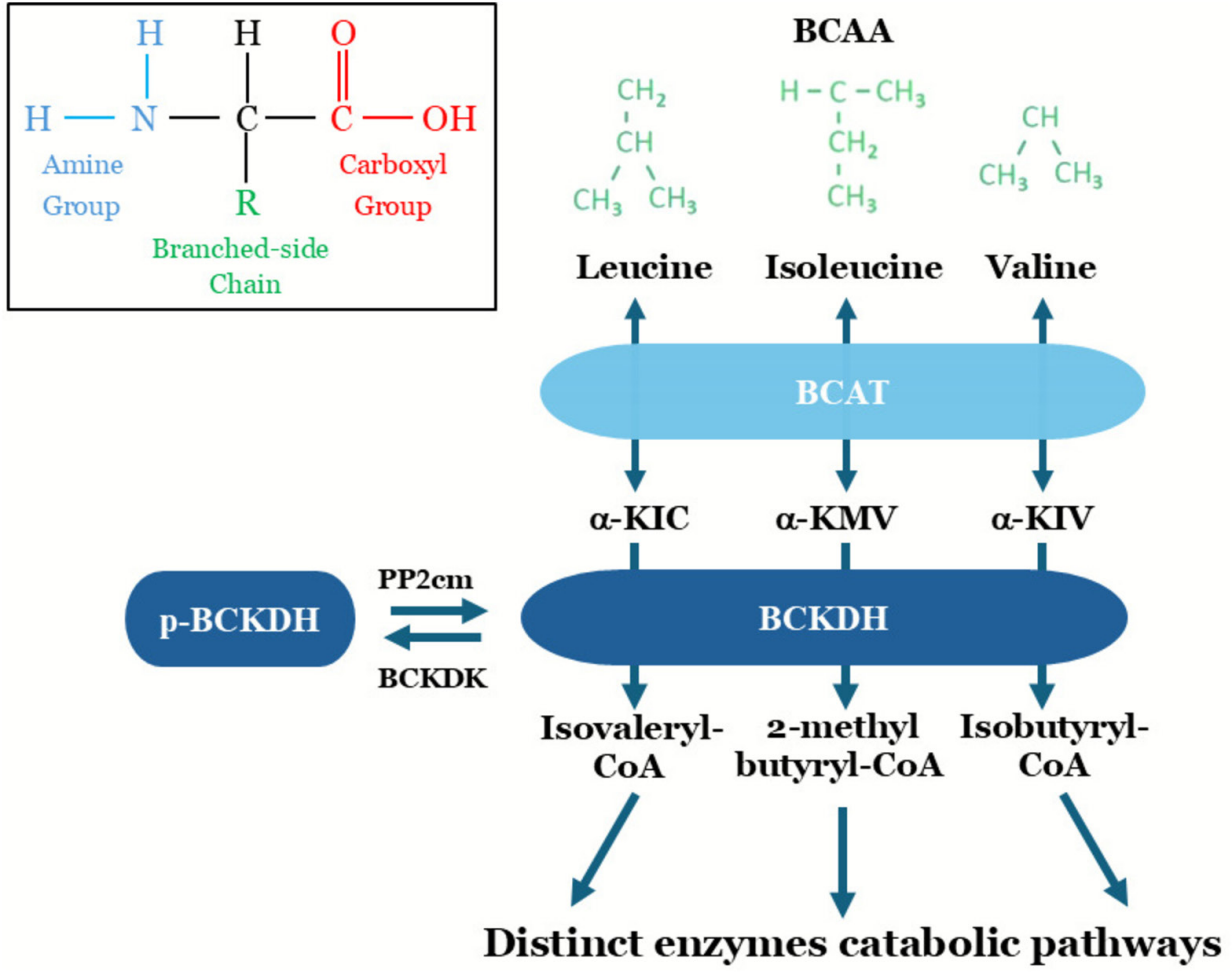
Initial common steps of branched‐chain amino acids (BCAA) catabolism. BCAA, branched‐chain amino acid; BCAT, branched‐chain aminotransferase; α‐KIC, alpha ketoisocaproate; α‐KMV, alpha ketomethylvalerate; α‐KIV, alpha ketoisovalerate; BCKDH, branched‐chain ketoacid dehydrogenase; p‐BCKDH, phosphorylated branched‐chain ketoacid dehydrogenase; BCKDK, BCKDH kinase; PP2cm, BCKDH phosphatase.

A divergence in each BCAA catabolic pathway begins after these two initial steps (Figure 1). The products of the BCKDH oxidative decarboxylation reaction**—**the branched‐chain acyl‐CoA derivatives**—**undergo oxidation through different dehydrogenases, resulting in leucine as a ketogenic **AA**, as it will produce acetyl‐CoA and acetoacetate, while valine is glucogenic, in that it is converted to succinyl‐CoA**—**an intermediate of the Krebs cycle. Isoleucine is metabolized to succinyl‐CoA via methylmalonyl‐CoA and also acetyl‐CoA and can therefore be considered a glucogenic and ketogenic **AA** [24].

Roughly 100 years after the first BCAA was described, isolation of insulin was made by Banting and Best [25], and the association of obesity with its unproper activity has been recognized in the last decades [26, 27, 28]. Insulin resistance is the terminology used to characterize any state to which responsiveness to insulin is lower than normal [29]. Beyond hyperinsulinemia itself, insulin resistance can be influenced by numerous factors, such as aging, weight, ethnic characteristics, nutritional status, physical activity and exercise, dietary habits, energy intake, gut microbiota, and medications. The insulin classical recognized action is the enhancement of glucose utilization and its adequate storage aiming to defend the organism against hyperglycemia negative effects, but in high levels, it consequentially promotes white adipose tissue expansion and lipogenesis in the liver, leading to hepatic steatosis and hyperlipidemia [30].

Insulin signaling influences not only the classical insulin‐sensitive organs and tissues but all of the body system, as augmented insulin circulating levels can downregulate insulin receptors and desensitize postreceptor pathways in cells, especially in leukocytes [26]. Excess of nutrient ingestion can increase the levels of insulin and also the concentration of circulating BCAAs, consequentially activating the mammalian target of rapamycin complex (mTORC1) that can further inhibit insulin receptor/insulin receptor substrate (IRS) signaling [17]. In this context, BCAAs are viewed not only as substrates for protein and peptide synthesis but as signaling modulators regulating the main body metabolism of glucose, lipids, and proteins as well, via the PI3K/AKT/mTOR signaling pathways [23]. Chronic mTORC1 stimulus also exacerbates the endoplasmic reticulum stress response by turning on an unfolded protein response that can exacerbate insulin resistance [31]. Thereby, two mechanisms have been proposed to explain the role of BCAAs in the insulin resistance phenotype: first, overactivation of mTORC1 by BCAAs; and second, the dysmetabolism model, which proposes that the accumulation of BCAA‐derived metabolites causes mitochondrial dysfunction in β cells and adipocytes [23].

The energy derived from caloric excess can also be stored in adipocytes or as ectopic lipid forms in myocytes, beta‐pancreatic cells, hepatocytes, and other cell types, leading to obesity and T2D [29]. Even the adipocyte, as the designed cell for this function, can be negatively affected by the accumulation of energy excess [32]. Also, increased visceral adiposity creates a microenvironment conducive to inflammation, characterized by tissue hypoxia, adipocyte death, and dysregulated adipokine secretion, including decreased adiponectin levels and increased production of leptin, resistin, retinol‐binding protein 4, interleukin‐6 (IL‐6), interleukin‐8 (IL‐8), monocyte chemoattractant protein‐1, and granulocyte colony‐stimulating factor [33, 34]. Together with other cytokines, these factors can attract the phenotypic classic activated macrophages (named as M1 macrophages), which release more inflammatory factors such as tumoral necrose factor (TNF‐α) that may have local and systemic phlogistic effects inducing insulin resistance [35]. Mechanistically, these proinflammatory cytokines M1‐macrophages derived create insulin resistance through suppressors of cytokines signaling (SOCS1 or SOCS3), targeting the phosphotyrosinated IRS1 or IRS2 for ubiquitination through a BC‐containing ubiquitin ligase E3 and consequentially degradation [36]. IRS1 and IRS2 can also undergo poly‐ubiquitinoylation during inflammation, chronic nutrient excess, or hyperinsulinemia through other mechanisms [37]. As most IRS1/2 phosphorylation of serine/threonine is derived from the PI3K/Akt/mTOR cascade during insulin stimulation, it indicates that IRS1/2 phospho‐serine/threonine is likely a feedback status that occurred during a chronic insulin stimulation [17, 38], linking hyperinsulinemia, chronic inflammation, and insulin resistance.

Although chronic high plasma level of BCAA has been described as a predictive factor for insulin resistance and T2DM, and its augment can be observed even 10 years earlier than the deterioration of glucose tolerance [4]; it is still not clear if BCAAs are a causative factor in insulin resistance and T2D or a consequence biomarker of diminished insulin capacity function [39, 40]. The same situation occurs with inflammatory factors, and more recently BCAA and its derivatives have been implicated in the modulation of leukocytes in immunometabolism studies which turned on the light to the relation of BCAA and inflammation [41, 42]. Importantly, the detrimental effects of BCAAs on insulin resistance were mostly noticed in high‐fat diet (HFD)–fed and/or obese situations [43]. Thereby, our aim in this narrative review is to describe and present the relation between BCAA and inflammation in the obesity and diabetes context.

## 2 HFD and Obesity/Diabetes Models Interference in the BCAA Metabolism

### 2.1 BCAA Catabolism Enzyme Levels

HFD, obesity (ob/ob), or type 2 diabetic (db/db) animal models have been demonstrated to alter systemic [44] and specific cellular processes, including BCAA catabolism and the immunometabolism [45, 46]. As aforementioned, the first enzyme in the BCAA catabolism pathway is the BCAT, which is poorly posttranslational regulated in comparison to BCDKH [19, 47]. Thus, the most studied aspect is the tissue levels of the enzymes BCKDK and the PP2cm that tightly modulated the most relevant factor in the activity of the rate‐limiting enzyme BCKDH: its phosphorilation status [21, 22].

In liver tissue, studies evaluating HFD protocols to wild‐type (WT) mice or a normal diet (ND) to diabetic (db/db, and T2D streptozotocin‐induced) and obese animal models (ob/ob) presented unequivocally reduced mRNA and/or protein levels of PP2cm [48, 49, 50, 51]. A statistical increase in the mRNA and protein levels of the BCKDK was seen in all studies, except in the Gart [50] results (Table 1). In the research, the Ldlr−/−Leiden mice, an established experimental model for diet‐induced Nonalcoholic Fatty Liver Disease/nonalcoholic steatohepatitis (NAFLD/NASH) with atherosclerosis, when treated 26 weeks with 41% fat and high in fructose HFD, presented a decreased mRNA levels of BCAT and PP2cm and although the BCKDK did not statiscally change, it presented a tendency to increase (*p* = 0.062). The mRNA levels of the BCKDH enzyme in this particular study were unchanged [50]. Two other studies analyzed the liver tissue levels of expression of the BCKDH. An HFD was provided to C57BL/6J or outbred male CD‐1 mice augmenting the expression level of BCKDH [32, 52]. Although it is known that the levels of an mRNA do not always correspond to the actual protein levels and activity, this might correspond a compensatory mechanisms in these cases and can be corroborated by the lack of alteration found in the blood BCAA levels in some studies (see 2.3 Plasma BCAAand BCKA [branched‐chain keto acids]).

**TABLE 1:** High‐fat diet and obesity/diabetes models interference in BCAA degradation enzyme levels, expression and activity.

Ref.	Animal (evaluation age)	Cell/tissue analyzed	Characteristics of the protocols	mRNA expression and enzymes levels in tissue analyzed	BCKDH enzyme activity
Gart et al. [50]	Male *Ldlr−/−* Leiden mice. Genetic background: 94% C57BL/6J (38wo)	LT	ND or 41% fat and 44% fructose HFD, 26w	HFD LT: < mRNA Bcat2, PP2cm (*p* < 0.05) & > Bckdk (*p* = 0.062) mRNA Bckdha and Bckdhb, no diff.	> p‐BKCDH/BKCDH (*p* < 0.05)
Lian et al. [49]	Male ob/ob and male STZ‐induced T2D C57BL/6 (11wo)	eWAT, LT, SMT	ND, 6‐10w	ob/ob eWAT: < PP2cm (*p* < 0.01) & > Bckdk (*p* < 0.01) ob/ob LT: < PP2cm (*p* < 0.01) & > Bckdk (*p* < 0.01) ob/ob SMT: >Bckdk (*p* < 0.01). PP2cm, no diff. STZ T2D eWAT: < PP2cm (*p* < 0.01) & > Bckdk (*p* < 0.05) STZ T2D LT: < PP2cm (*p* < 0.01) & > Bckdk (*p* < 0.01) STZ T2D SMT: >Bckdk (*p* < 0.01). PP2cm, no diff.	ob/ob eWAT: < BKCDH activity (*p* < 0.05) & > p‐BKCDH/BKCDH (*p* < 0.01) ob/ob LT: < BKCDH activity (*p* < 0.05) & > p‐BKCDH/BKCDH (*p* < 0.01) ob/ob SMT: < BKCDH activity (*p* < 0.01) & > p‐BKCDH/BKCDH (*p* < 0.01) STZ T2D eWAT: < BKCDH activity (*p* < 0.05) & > p‐BKCDH/BKCDH (*p* < 0.01) STZ T2D LT: < BKCDH activity (*p* < 0.05) & > p‐BKCDH/BKCDH (*p* < 0.05) STZ T2D SMT: < BKCDH activity (*p* < 0.01) & > p‐BKCDH/BKCDH (*p* < 0.01)
Liu et al. [51]	Male C57BLKS db/db (14wo)	LT, SMT, KT, Peritoneal M0	ND, 4w	db/db LT: < mRNA PP2cm (*p* < 0.05) & > mRNA Bckdk (*p* < 0.01) db/db SMT: < mRNA Bcat2, Bckdhb, Bckdk, PP2cm (*p* < 0.01) db/db KT: < mRNA Bckdha, PP2cm (*p* < 0.05) & > mRNA Bckdk (*p* < 0.05) db/db M0: mRNA Bcat 1/2, Bckdha, Bckdhb, Bckdk and PP2cm, no diff.	db/db LT: > p‐BKCDH/BKCDH (*p* < 0.001) db/db SMT: > p‐BKCDH/BKCDH (*p* < 0.05) db/db KT: > p‐BKCDH/BKCDH (*p* < 0.05) db/db M0: no diff.
Zhang et al. [48]	Male C57BL/6J (20‐22wo)	eWAT, LT, SMT	ND or 60% fat HFD, 12w	HFD eWAT: < PP2cm (*p* < 0.05) & > Bckdk (*p* < 0.05) HFD LT: < PP2cm (*p* < 0.05) & > Bckdk (*p* < 0.05) SMT: Bckdk no diff. PP2cm not evaluated	HFD eWAT: < BKCDH activity (*p* < 0.05) & > p‐BKCDH/BKCDH (*p* < 0.05) HFD LT: < BKCDH activity (*p* < 0.05) & > p‐BKCDH/BKCDH (*p* < 0.05) HFD SMT: no diff.
Burril et al. [32]	Male C57BL/6J (16‐19wo)	eWAT, iWAT, BAT, LT, SMT	ND or 60% fat HFD, 12‐15w	HFD eWAT: < mRNA Bcat2, Bckdha, Bckdhb (*p* < 0.05) HFD iWAT: < mRNA Bcat2, Bckdhb (*p* < 0.05). Bckdha no diff. BAT: Bcat2, Bckdha, Bckdhb no diff. HFD LT: > mRNA Bckdhb (*p* < 0.05). Bcat2 no diff. SMT: Bcat2, Bckdha, Bckdhb no diff.	—
Li et al. [52]	Male outbred CD‐1 mice (22wo)	eWAT, LT, PT	ND or 42% fat HFD, 17w	HFD eWAT: < mRNA BCAA degradation enzymes (*p* < 0.05) HFD LT: > mRNA BCAA degradation enzymes (*p* < 0.05) HFD PT: > mRNA BCAA degradation enzymes (*p* < 0.05)	—
Huang et al. [53]	Male C57BL/6 (24wo)	ATM	ND or 60% fat HFD, 16w	HFD ATM: < mRNA and protein levels of Bcat1 and Bckdk (*p* < 0.05). > PP2cm protein level (*p* = 0.03). mRNA Bcat2, Bckdha, PP2cm, no diff.	< p‐BKCDH
Zhao et al. [54]	Male WT and *ApoE−/−* C57BL/6J(22wo)	Peritoneal M0	ND or 40% fat +1.25% CHOL HFD, 14w	HFD *ApoE−/−* M0: < mRNA Bcat2 (< 0.01)	> p‐BKCDH/BKCDH (*p* < 0.01)

Abbreviations: ApoE: apoliprotein E; ATM: adipose tissue macrophage; BAT: brown adipose tissue; eWAT: eppididimal white adipose tissue; HFD: high‐fat diet; iWAT: inguinal white adipose tissue; LDL: low density lipoprotein; LT: liver tissue; M0: macrophage; ND: normal diet; PT: pancreas tissue; ST: skeletal muscle tissue; STZ: streptozotocin; w: weeks; wo: weeks‐old.

In the white adipose tissue, an up‐regulation of the inhibitory enzyme BCKDK, lower PP2cm protein levels, and a decrease in expression levels of BCAA degradation enzymes (*p* < 0.05) were unequivocally seen in the studies (Table 1). C57BL/6J and outbred males CD‐1 mice were treated with an HFD comprising 40%–60% calories of fat for a period of 10–18 weeks [32, 48, 52] and ob/ob and T2D streptozotocin‐induced C57BL/6 mice with a ND [49]. Interestingly, no significant expression or minor alterations were seen in the main BCAA catabolism enzymes in brown adipose tissue [32].

In adipose tissue–resident macrophages of C57BL/6 mice fed a 60% HFD, BCAT1 and BCKDK presented lower mRNA and protein levels. PP2cm mRNA expression showed only a tendency to increase but was not significant, although its protein levels were augmented (*p* = 0.03) [53]. BCAA degradation enzyme expression by peritoneal macrophage of HFD‐fed mice and diabetic models are yet contradictory. In Zhao and colleagues' study, HFD‐fed versus ND‐fed C57BL/6 ApoE^−/−^ animals presented BCAT2, BCKDHA, and PP2cm mRNA reduced expression [54]. Although when comparing this cell type of db/db with WT mice, the study of Liu and colleagues presented no significant difference in the gene expression of key genes involved in BCAAs catabolism (Slc7a5, Bcat1, Bcat2, Bckdha, Bckdhb, Bckdk, Dbt, and Pp2cm) [51].

The skeletal muscle tissue presented also some contradictory results depending on the study design. In Burril and Zhang's studies, the level of muscle PP2cm and BCKDK did not alter, although Lian and Liu's studies presented a significant increase and decrease in BCKDK protein and mRNA levels, respectively [32, 48, 49, 51]. Interestingly in the Liu study, distinctly from the Lian study, there was a decrease in the PP2cm mRNA levels [49, 51].

### 2.2 BCAA Degradation Enzyme Activity

In addition to assessing the gene expression and protein levels of the BCKDH enzyme and its regulators, measuring its actual activity is also an important form of evaluation. Naturally, it reflects in part the activity of the PP2cm and BCKDK enzymes that were already seen are distinctly modulated in the tissues. Here, the forms of assessment are the measurement of reaction products carried out by BCKDH and the inactive/phosphorylated p‐BCKDH E1α subunit per total BCKDH ratio, as the immunoreactivity of the BCKDH pSer293 antibody is directly correlated with BCKDH activity [21].

In the liver tissue, increased levels of p‐BCKDH, which is related to a decreased activity of this enzyme, were seen in C57BL/6J and Ldlr−/−.Leiden mice in an HFD feeding protocol [48, 50] or in obese or diabetes models in a ND protocol (ob/ob; STZ‐induced T2D C57BL/6; db/db) (Table 1) [49, 51]. These results reflect the aforementioned alterations in these tissue levels of PP2cm and BCKDK regulatories enzymes of BCKDH. Although, in the study of Gart and colleagues, this did not translate into alteration in the blood BCAA levels [50].

In the white adipose tissue, higher levels of p‐BCKDH and consequently a lower activity of the BCKDH were seen in either a wild type mice in an HFD protocol [48], in ob/ob, or in T2D streptozotocin‐induced ND‐fed animals (Table 1) [49]. As the only study from adipose tissue macrophage, Huang and colleagues presented an increase in the level of PP2cm and a decrease in BCKDK, reflected by less p‐BCKDH in C57BL/6 mice fed with an HFD (Table 1) [53]. Although that, the BCKDH total content remained unaltered. This modulation could represent some compensatory mechanism for the BCAT diminished levels found, as the intracellular BCAA/BCKA ratio does not change expressively, although the levels of the molecules increased almost two‐fold.

In peritoneal macrophages, distinct results are seen. Zhao and colleagues showed that an HFD significantly augmented levels of the inactive p‐BCKDH in C57BL/6 peritoneal macrophages (Table 1) [54]. Conflicting to this study, no significant differences between the WT and db/db group peritoneal macrophages protein levels of p‐BCKDH were seen in the Liu study, reflecting the aforementioned lack of alteration in the gene expression of its regulatory enzymes (Table 1) [51]. A difference here can be seen in relation to the protocols utilized, Zhao and colleagues analyzed the HFD‐fed male C57BL/6 ApoE−/− derived peritoneal macrophage just after its isolation, while Liu and colleagues analyzed ND‐fed C57BLKS/Leprdb after incubation of these cells in RPMI medium by some time.

In the skeletal muscle discrepant results were seen. The ND protocol in obese or diabetes models increased the levels of p‐BCKDH and the proportion of p‐BCKDH/BCKDH in the Lian and Liu study (Table 1) [49, 51]. Otherwise, an HFD feeding protocol presented no significant effect upon BCKDH phosphorylation or activity in skeletal muscle in C57BL/6 animals (the levels of PP2cm and BCKDK were not analyzed) (Table 1) [48].

### 2.3 Plasma BCAA and BCKA

Chronic high plasma levels of BCAA have been described as a major consequence of its reduced catabolism in obese and T2D situations [39]. Importantly, HFDs also have been demonstrated to impact systemic and cellular processes including altered BCAA catabolism [44, 45, 46]. Corroborating this, the majority of the animal studies evaluating this aspect confirmed it (Table 2).

**TABLE 2:** High‐fat diet and obesity/diabetes modulation of BCAA blood concentration.

Ref.	Animal (evaluation age)	Characteristics of the protocols	Results
Huang et al. [53]	Male C57BL/6 (24wo)	ND or 60% fat HFD, 16w	HFD: > BCAA & > BCKA (*p* < 0.01)
Lian et al. [49]	1‐ Male ob/ob and STZ‐induced T2DM C57BL/6 2‐ Male C57BL/6 (11wo)	1‐ND 2%–45% fat HFD, 4w	1‐ ob/ob ND: > BCAA & BCKA (*p* < 0.01) 1‐ STZ T2D ND: > BCAA & BCKA (*p* < 0.01) 2‐ C57BL/6 HFD: BCAA & BCKA no diff.
Takahashi et al. [55]	Male C57BL/6J (25‐26wo)	ND or 60% fat HFD, 18w	HFD: > L (*p* < 0.009) & > V (*p* < 0.02)
Wang et al. [56]	Male C57BL/6J (14wo)	ND or 60% fat HFD, 10w	HFD: > L (*p* < 0.05) & > V (*p* < 0.01)
Zhang et al. [48]	Male C57BL/6J (20‐22wo)	ND or 60% fat HFD, 12w	HFD: > BCAA & > BCKA (*p* < 0.05)
Zhao et al. [54]	Male *ApoE−/−* C57BL/6J (22wo)	ND or 40% fat +1.25% CHOL HFD, 14w	HFD *ApoE−/−*: > BCAA& > BCKA (*p* < 0.01)
Gart et al. [50]	Male *Ldlr−/−* Leiden mice. Genetic background: 94% C57BL/6J and 6% 129S (38wo)	ND or 41% fat and 44% fructose HFD, 26w	No diff.
Gong et al. [58]	Male db/db (12, 16, 20wo)	ND	db/db: > BCAA (*p* < 0.05)
Liu et al. [51]	Male C57BLKS db/db (14wo)	ND	db/db: > KMV (*p* < 0.01). BCAA: no diff.

Abbreviations: ApoE: apolipoprotein E; BCAA: branched‐chain amino acids; BCKA: branched‐chain keto acids; CHOL: cholesterol; HFD: high‐fat diet; KMV: keto methylvalerate; LDL: low density lipoprotein; ND: normal diet; STZ: streptozotocin; T2D: type‐2 diabetes mellitus; V: valine; w: weeks; wo: weeks‐old.

HFDs for a period of 10 to 18 weeks, elevated the BCAA and/or BCKA plasma levels of C57BL/6, ob/ob, and streptozotocin‐induced T2D animals [48, 49, 53, 54, 55, 56]. Contradicting these, only results from Gart and one protocol from the Lian study presented no difference in BCAA or BCKA plasma levels [49, 50]. However, here there are some aspects to consider the following: (i) The animals in the Gart study were male Ldlr^−^/^−^ Leiden mice with a genetic background of 94% C57BL/6J and 6% 129S, rendering the study somehow distinct from the others. Analyzing the only study evaluating plasma BCAA levels in an animal distinct from the C57BL/6 background in our search, it is understood that the time for the appearance of higher BCAA blood levels can be specie/breed‐dependent, as male Sprague–Dawley rats treated for only 2 weeks with a 45% fat HFD presented higher BCAA plasma levels after this short time of intervention [57]. (ii) In the Lian protocol with C57BL/6, mice were subjected to a shorter feeding duration in relatively young animals compared to the other studies, 4 weeks of a 45% fat HFD treatment in 7‐week‐old animals [49]. As already known, the impact of HFD in BCAA catabolism emerges concomitantly with aging [52]. Moreover, these factors can be considered limitations when interpreting the results of the described studies. Interestingly, in both studies, although plasma BCAA levels did not present a significant difference, the activity of BCKDH had significantly decreased in the tissues of the animals [49, 50].

The two studies evaluating the T2D (C57BLKS/Leprdb—db/db) mice under an ND feeding presented conflicting results [51, 58]. In the Liu and colleagues study the plasma BCAA levels of 14‐weeks‐old animals remained significantly unaltered in C57BLKS/Leprdb compared with its WT counterparts (C57BLKS) and the only BCAA catabolism product altered in this particular study, was an increase in KMV (BKCA) plasma levels in db/db group [51]. Evaluating the same animal and feeding protocol, Gong and colleagues presented that BCAA levels were significantly increased in the plasma of the db/db group in all three time points evaluated by their study (12, 16, and 20wo) [58]. Here, unlike what was observed in the HFD intervention, the time required to detect alterations in blood BCAA levels does not appear to be the determining factor. In Liu and colleagues' study, they evaluated this parameter in animals with 14‐week‐old whereas Gong and colleagues observed a significant difference as early as 12 weeks of age.

**FIGURE 2:**
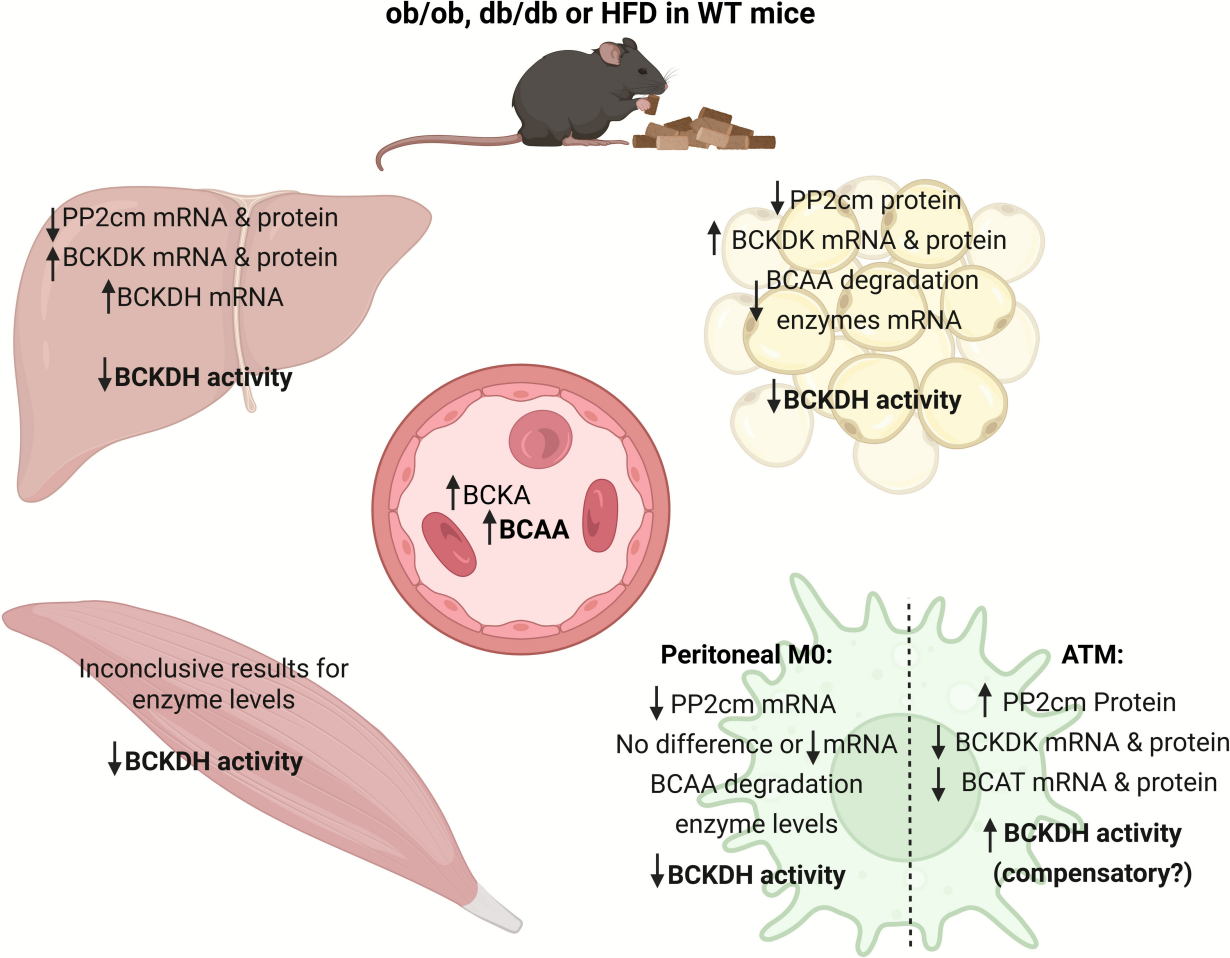
Overview of cell/tissue branched‐chain amino acids metabolism in obesity and diabetes‐induced animal models. BCAA, branched‐chain amino acid; BCKA, branched‐chain keto acids; BCAT, branched‐chain aminotransferase; BCKDH, branched‐chain ketoacid dehydrogenase; WT, wild‐type; BCKDK, BCKDH kinase; PP2cm, BCKDH phosphatase; ATM, adipose tissue macrophage; M0, macrophage; mRNA, messenger ribonucleic acid.

The complex interplay between BCAA metabolism and metabolic health is a subject with great relevance in the current scientific world. Recent studies in the field have presented roles for microbiota, polymorphism, and other alterations that culminate in higher circulating BCAA levels [2, 59, 60]. However, the BCAA catabolism enzyme regulation remains the most studied and important aspect. The collective evidence here described underscores that BCAA catabolism in the liver and white adipose tissue of mice is usually suppressed in HFD and metabolic‐inflammatory disease models such as obesity and type 2 diabetes where the circulating BCAA levels are generally high (Figure 2). As presented, this phenomenon is derived from disruptions in the expression and/or activity of key metabolic BCAA enzymes, such as the BCAT, BCKDH, BCKDK, and PP2cm [21] and corroborates with Heman and colleagues' study where the transplantation of a healthy adipose tissue into a BCAA catabolism defective mice by BCAT deletion reversed the augmentation of the circulating BCAA levels in the acceptor animals [61].

Although this pattern occurs in the liver and white adipose tissue, the catabolic impairment in other tissues varies significantly depending on the context (diet, animal/experimental model, cell type, age, etc.). One example is the skeletal muscle tissue where contradictory results are seen depending on the study design. In Burril and Zhang's studies, the level of muscle enzymes did not alter [32, 48], although, in the Lian and Liu studies BCKDK protein and mRNA level alterations were seen [49, 51]. Interestingly in the Liu et al. [51] study, although a decrease in BCKDK protein level occurred, there was also a decrease in the PP2cm mRNA levels resulting in a lower final activity of BCKDH and degradation of BCAA as in Lian study [49, 51]. This brings the possibility that the experimental model was the divergent factor, as the Burril and Zhang studies evaluated C57BL/6J in a 60% HFD protocol, and Lian and Liu worked with obese and diabetic models in ND protocols. Another point that needs further investigation in the field is the possible difference in BCAA catabolism between genders, as the studies described used male animals and human studies have demonstrated difference in plasma levels concentrations [4, 5].

## 3 BCAA‐Associated Inflammation Modulation

### 3.1 In Vitro and Ex Vivo Studies

BCAA's impact on in vitro and animal metabolism has been extensively researched for decades [19, 62, 63] and although BCAAs have a long history in basic science research, they have recently been implicated in the modulation of leukocyte phenotypes [41, 42]. With the evolution and improvement of immunometabolism techniques more studies have correlated the BCAA and inflammation relation (Table 3).

**TABLE 3:** Ex vivo, in vitro*,* and in vivo BCAA supplementation and modulation of inflammatory mediators.

Ref.	Model (evaluation age)	Cell/Tissue analyzed	Characteristics of the protocols	Results
Zhenyukh et al. [41]	Ex vivo	Healthy human donors PBMCs in RMPI‐1640 (0.38 mM of L and I and 0.17 mM of V as standard composition)	BCAA supplementation (4; 6; 8; 10; 12 mM) + stimulus: 30 ng/mL TNFα, 102 U/mL IL‐6 and or 30 mmol/L of glucose for 1 h	10 mM BCAA: >NF‐κB pathway activity (*p* < 0.01) and mRNA levels of IL‐6 (*p* < 0.01), TNFα (*p* < 0.01), ICAM‐1 (*p* < 0.01) and CD40L (*p* < 0.01)
Liu et al. [51]	Ex vivo	Peritoneal M0 and BDMD from WT mice in RPMI‐1640 (0.38 mM of L and I and 0.17 mM of V as standard composition)	BCAA supplementation (0.5; 10 mM) for 24 h	0.5 mM BCAA peritoneal and BMDM: mRNA levels of IL‐1β, IL‐6, TNFα and iNOS, no difference 10 mM BCAA for BMDM: mRNA levels IL‐1β and iNOS, no difference
Bonvini et al. [65]	In vitro	RAW 264.7 macrophages in DMEM (0,8 mM of each BCAA as standard composition)	L 1.2 mM or I 1.2 mM or V 1.2 mM or LIV 3.6 mM (1.2 mM of each) plus LPS (1 μg/ML) for 24 or 48 h	L + LPS: > NO production (*p* < 0.05). No difference IL‐6, TNF‐α and PGE2 levels I + LPS: > NO production (*p* < 0.05) & IL‐6 (24 h pre or 24 h pre and during LPS treatment) (*p* < 0.05). No difference in TNF‐α and PGE2 levels. V + LPS: > NO production (*p* < 0.05). No difference in IL‐6, TNF‐α and PGE2 level LIV + LPS: no difference in NO, IL‐6, TNF‐α and PGE2 levels
Gong et al. [58]	In vitro	Mio‐M1 cells in DMEM/F12 (0.4–0.45 mM of each BCAAs as standard composition)	HG without BCAA, HG + low BCAA (2 mmol/L) and HG + high BCAA (5 mmol/L) for 24 h. *NG = 0.5 mmol/L D‐glucose, HG = 25 mM D‐glucose	HG + high BCAA: > mRNA TNF‐α and protein levels
Zhao et al. [54]	In vitro	RAW 264.7 macrophages in personalized DMEM/HG (0.2 mM of L and 0.1 of I and V)	BCAA supplementation (3.6 mM) for 12 h	BCAA: > IL‐1β and TNF‐α levels mRNA and protein levels (*p* < 0.01)
Gart et al. [50]	Male *Ldlr−/−* Leiden mice. Genetic background: 94% C57BL/6J and 6% 129S. (38wo)	LT	ND or 41% fat and 44% fructose HFD, 26w V or I supplementation (2‐3 mg/g/day BW), in the last 12w	V or I: < mRNA levels IL‐1, IL‐1a, IL‐1b, IL‐2, IL‐3, IL‐4, IL‐5, IL‐6, IL‐13, IL‐15, IL‐33, IFNG, CSF1, CSF2, CSF3, OSM, TNFA, CCR2, and CXCL12 (*p* < 0.05), but not its protein levels. I: <mRNA levels CCL‐2; CXCL‐1and CXCL‐2 protein levels (*p* < 0.05).
Terakura et al. [68]	Male C57BL/6 db/db (41wo)	LT, WAT	ND or ND + BCAA (2–3 mg/g/day BW), 36w	BCAA LT: < protein levels IL‐1β, IL‐6, IL‐18 and TNF‐α (*p* < 0.05) BCAA WAT: < M0 infiltration and mRNA levels of IL‐6, TNF‐α and MCP‐1 (*p* < 0.05).
Feijó et al. [69]	Male Wistar rats (31 wo)	Hippocampus	ND or 45% fat HFD + 230 mM *ad libitum* sugary solution, 19w BCAA gavage (0,750 mg/g/day BW), in the last 4w	BCAA: < IL‐1 β immunoreactivity (*p* < 0.05)
Zhang et al. [48]	Male C57BL/6J (20–22 wo)	LT	ND and 60% fat HFD with or without BCAA in drinking water (7–9 mg/g/day BW), 12w	BCAA: >mRNA levels TNF‐α, IL‐6, IL‐1β, and MCP‐1 (*p* < 0.05)
Liu et al. [51]	Male C57BLKS db/db (14wo)	Blood, LT, KT	ND with or without BCAA gavage (1.5 mg/g/day BW), 4w	BCAA blood: > blood IL‐6 (*p* < 0.05). BCAA LT: > mRNAs levels of IL‐ 1β and IL‐6 and IL‐1β protein (*p* < 0.05) BCAA KT: > mRNAs levels of IL‐ 1β, IL‐6, MCP‐1 (*p* < 0.01) and proteins from IL‐1β and IL‐6 (*p* < 0.01)
Felicianna [66]	Male C57BL/6J (17 wo)	Blood, LT, WAT and Hypothalamus	42%fat +0.2%chol HFD or HFD with voluntary oral V supplementation (0.062 mg/g/day BW), 9w	V blood: no diff. V LT: < protein levels TNF‐α, IL‐6 (*p* < 0.05) V WAT: < mRNA levels IL‐10 (*p* < 0.05), TNF‐α (*p* < 0.01) and TGFb1 (*p* < 0.0001) V Hypothalamus: < protein levels IL‐6 (*p* < 0.05)
Huang et al. [53]	Male C57BL/6 db/db (24 wo)	WAT	60% fat HFD, ND or a 150% added BCAA diet (17.0 mg/g/day BW), 16w	BCAA WAT: > protein levels IL‐1β, TNF‐α and MCP‐1 (*p* < 0.0001)

Abbreviations: ApoE: apoliprotein E; BCAA: branched‐chain amino acids; BMDM: bone marrow derived macrophage; CCL‐2: C‐C Motif Chemokine Ligand 2; CCR2: C‐C Motif Chemokine Receptor 2; CD40L: Cluster Of Differentiation 40 Ligand; CSF1: colony stimulating factor 1; CSF2: colony stimulating factor 2; CSF3: colony stimulating factor 3; CXCL1: C‐X‐C Motif Chemokine Ligand 1; CXCL‐2: C‐X‐C Motif Chemokine Ligand 2; CXCL12: C‐X‐C Motif Chemokine Ligand 12; DMEM: Dulbecco's modified Eagle medium; HFD: high‐fat diet; HG: high glucose; ICAM‐1: Intercellular Adhesion Molecule‐1; IFNG: interferon gamma; IL‐1: interleukin‐1; IL‐1a: interleukin‐1 alpha; IL‐1b: interleukin‐1 beta; IL‐2: interleukin‐2; IL‐3: interleukin‐3; IL‐4: interleukin‐4; IL‐5: interleukin‐5; IL‐6: interleukin‐6; IL‐13: interleukin‐13; IL‐15: interleukin‐15; IL‐18: interleukin‐18; IL‐33: interleukin‐33; I: isoleucine; iNOS: Inducible Nitric Oxide Synthase; L: leucine; LDL: low density lipoprotein; LPS: lipopolysacharides; LT: liver tissue; M0: macrophage; MCP‐1: monocyte chemoattractant protein‐1; NG: normal glucose; NF kB: nuclear factor kappa B; NO: oxide nitric; OSM: oncostatin M; PBMC: peripheral blood mononuclear cells; PGE2: prostaglandin E2; RPMI: Roswell Park Memorial Institute medium; TGFb1: transforming growth factor beta; TNFa: tumor necrose factor alpha; V: valine; w: weeks; wo: weeks‐old; WT: wild type.

Through ex‐vivo use of several inhibitors and activators of molecular pathways, Zhenyukh and colleagues revealed that the activation of mTORC1 by BCAA is associated with the production of reactive oxygen species (ROS) and mitochondrial dysfunction. Furthermore, 10 mM of BCAAs (approximately 50–100 times the normal physiological levels), added as a single stimulus to RPMI medium (containing 0.38‐mM leucine and isoleucine, 0.17‐mM valine, and 5.5 mmol/L glucose) for 1 h, was capable of activating transcription factor nuclear factor κB (NF‐κB) in cultured PBMCs from human healthy donors. This activation resulted in a two‐fold or greater release of pro‐inflammatory molecules, including IL‐6, TNFα, ICAM‐1, and CD40L. When evaluating the activity of NF‐κB by its p65 component phosphorylation, 10 mM of BCAA treatment showed to augment these parameters comparable to LPS (1 μg/mL). BCAA concentrations ranging from 3 to 12 mM exhibited oxidative effects similar to treatments with 30 ng/mL TNFα, 102 U/mL IL‐6, or 30 mmol/L glucose—conditions typically observed in obesity, type 2 diabetes, and related clinical scenarios [41]. Contradicting this data, Liu and colleagues reported that 24 h of BCAA treatment at high concentrations (10 mM) did not alter mRNA levels of pro‐inflammatory factors IL‐1β, Inducible Nitric Oxide Synthase (iNOS), mitochondrial ROS, or ATP production in BMDMs cultured ex‐vivo in RPMI‐1640 medium. Neither the considered higher physiological concentration of BCAA (0.5 mM) supplementation had significantly altered the production of pro‐inflammatory factors in M1‐like peritoneal macrophages or in M0 or M1‐like BMDM [51]. Thus, from our search, the isolated BCAA supplementation has only been proved to alter the metabolic activity of leukocytes in the particular ex vivo study of Zhenyukh [41] and every other study, in vitro or experimental, had used some type of additive stimulus (directly or from HFD protocols) to verify the interference of BCAA in the leukocyte metabolism.

In vitro studies provide a simplified approach to analyzing the expression and levels of cytokines and chemokines, been an efficient method for understanding and describing the mechanistic effects of various treatment factors. To date, more than 40 in vitro assay models have been developed, with ROS (reactive oxygen species) and interleukin assays being among the most widely used to investigate the impact of specific factors [64].

Studies from Bonvini, Gong, and Zhao used classical inflammatory stimulus in cell culture to understand the additive BCAA action (Table 3) [54, 58, 65]. Bonvini and colleagues treated RAW 264.7 macrophage cells in a high glucose medium (25 mmol/L) with 1 μg/mL of LPS in distinct protocols using BCAAs (L 1.2 mM or I 1.2 mM or V 1.2 mM or LIV 1.2 mM of each AA). They demonstrated that NO production increased in all individual AA treatments, but it was unaltered in LIV. The IL‐6 supernatant concentrations increased compared to control when it was supplemented 24 h before and were supplemented 24 h pre and during the LPS treatment, but TNF‐α and prostaglandin E2 supernatant levels were unaltered in all treatments and protocols [65]. Gong and colleagues adding high levels of BCAA (5 mM) in Mio‐M1 cells (Moorfields/Institute of Ophthalmology‐Müller 1) cultured in a high glucose (25 mM) medium showed an increase in TNF‐α protein levels [58]. RAW 264.7 macrophages supplemented with BCAA (3.6 mM totalizing 4.0 mM) for 12 h also in a high glucose (25 mM) medium, resulting in increased IL‐1β and TNF‐α levels mRNA and protein levels [54].

### 3.2 Murine and Human Studies

As in BCAA metabolism‐focused studies (Section 2), diet manipulation and models of obesity or diabetes have been necessary to study the effects of the BCAA in inflammation modulation. Without a previous initial metabolism alteration, the isolated supplementation of moderate doses of BCAA appears to lack the capacity to alter the immune system activity of animals (Table 3).

Murine studies evaluating HFD protocols in wild‐type C57BL/6 present distinct results [48, 66]. A voluntary oral low‐dose valine supplementation (0.062 mg/g/day BW, 9w) in C57BL/6J with induced metabolic dysfunction‐associated steatosis liver disease mice fed with an HFD showed lower levels of hepatic TNF‐α and IL‐6 protein, and lower IL‐10, TNFA, TGFB mRNA levels in WAT [66]. Similarly, 12 weeks of valine or isoleucine supplementation (2‐3 mg/g BW/day, 26w) after a high‐fructose and HFD protocol in male Ldlr−/−.Leiden mice showed that valine and isoleucine (at greater effect) were capable of decreasing the mRNA levels of various immune hepatic genes, including IL‐1β, IL‐6, and TNF‐α, but interestingly it did not translate to the protein level [50]. Zhang and colleagues' study in wild‐type C57BL/6 animals contradicted the above results. Utilizing a high‐fat percentage (60%) HFD with or without a high dose of the tree BCAAs into the drinking water (L 25 g, I 12.5 g, V 12.5 g for 1 L, totalizing 9 mg/g/day of BW, 12w), they showed that the supplementation of BCAA atop HFD further increases plasma BCAA level and an upregulation of the expression of hepatic proinflammatory cytokines TNF‐α, IL‐6, IL‐1β, and MCP‐1. This occurred even with BCAA supplementation attenuation of the classical HFD‐induced weight gain. Interestingly, they showed that the chronic BCAA supplementation to animals feeding an ND does not significantly change plasma BCAA levels and does not alter body weight, plasma TG, glucose homeostasis, and liver structure/function [48]. Interestingly, this study used the higher dose of BCAA supplementation found in our search, and as stated by Jian and colleagues, it seems that different doses of BCAA supplementation could have opposite effects on liver tissue inflammation [67].

Murine diabetic animal models (db/db C57/BL6 mice), also presented contradictory results [51, 53, 68]. db/db animals feeding an ND and receiving BCAA supplementation (1.5 L:0.8I:1 V, 1.5 mg/g/day BW, 4w) in the Liu and colleagues study, increased the hepatic IL‐1β protein level and the mRNAs levels of IL‐ 1β and IL‐6, IL‐6 levels were also elevated in the blood of the animals [51]. Contrary to this, the BCAA supplementation (2–3 mg/g BW/day 2 L: 1I: 1.2 V proportion, 36w) along with an ND significantly decreased liver IL‐1β, IL‐6, IL‐18, and TNF‐α protein levels and WAT IL‐6, TNFA, and MCP‐1 mRNA levels along with a decreased macrophage infiltration in this tissue [68]. Also, a high BCAA supplementation (1.8 L:1I:1.3 V ≈ 17.0 mg/g/day BW, 16w) in the Huang and colleagues study increased the IL‐1β, TNF‐α and MCP‐1 protein levels in the WAT of db/db animals [53].

In a study evaluating the effect of BCAA in the central nervous system, 3‐months‐old male Wistar rats received a normal diet or 45% fat HFD plus a 42 g/L ad libitum sugary solution for 19 weeks and in the final 4 weeks they were supplemented with BCAA (0.750 mg/g BW). Isoleucine was the only BCAA to significantly increase (*p <* 0.02) in the central nervous system (measured in the cerebellum tissue) after the initial treatment with 45% fat HFD plus a 42 g/L ad libitum sugary solution and the 4 weeks supplementation with BCAA. At the cerebral cortex, there were just IL‐6 levels diet‐related increase with no effect difference with the BCAA supplementation. Although that, in the hippocampus, was shown that BCAA supplementation decreased IL‐1 β immunoreactivity (*p <* 0.05) in the HFD‐fed animal group [69].

In humans, several studies have investigated the relationship between BCAA blood levels and IL‐6 in pediatric populations with varying adiposity (Table 4) [70, 71, 72, 73]. Perng et al. reported a significant positive association between a BCAA‐related metabolite pattern, which included BCAA, aromatic AAs and related metabolites, and IL‐6 levels in both lean and with obesity children [71]. However, a follow‐up study by Perng et al. found no association between the same BCAA pattern and IL‐6 levels when comparing baseline and measurements taken 5 years later [70]. Similarly, Bugajska et al. did not find a significant correlation between BCAA and IL‐6 concentrations in lean and with overweight/obesity children [73]. In contrast, Cosentino et al. observed a significant positive correlation between BCAA and IL‐6 and BCAA and CRP (c‐reactive protein) levels in lean and with obesity adolescents [72], suggesting a potential age‐related or developmental differences in this metabolic‐inflammatory axis (Table 4).

**TABLE 4:** Human blood BCAA and inflammatory mediators levels.

Ref.	Population	Characteristics of the populations	BCAA and inflammatory markers correlation
Perng et al. [70]	Lean and with obesity children	Obesity: 32M/52F; mean age 8.2 ± 1.0 years; BMI z‐score 2.07 ± 0,29; fat mass 15.5 ± 5.1 kg; HOMA‐IR 3.49 ± 0.81 Lean: 84M/66F; mean age 7.9 ± 0.8 years; BMI z‐score −0.04 ± 0,72; fat mass 5.7 ± 1.6 kg; HOMA‐IR 1.65 ± 0.30	Positive between BCAA pattern (BCAA and aromatic amino acids an metabolites) and IL‐6. 0.09 per unit of factor score (CI 95%, 0.02–0.17)
Perng et al. [70]	Children	104M/109F; mean age 13.0 ± 0.7/13.1 ± 0.7 years; BMI 21.3 ± 5.0/23.5 ± 5.7; fat mass N.D.; HOMA‐IR 3.9 ± 3.7/4.3 ± 3.1	No correlation found between BCAA and IL‐6 levels
Bugajska et al. [73]	Lean and with overweight/obesity children	Overweight/obesity: 12M/8F; mean age 7.7 ± 2.3 years; BMI 26.8 ± 5.0; fat mass N.D.; HOMA‐IR N.D. Lean: 4M/8F; mean age 6.5 ± 2.2 years; BMI 14.8 ± 1.5; fat mass N.D.; HOMA‐IR N.D.	No correlation found between BCAA and IL‐6 levels
Cosentino et al. [72]	Lean and with obesity adolescents	Obesity: 3M/4F; mean age 15.9 ± 0.5 years; BMI z‐score 2.46 ± 0,16; fat mass 46.11 ± 2.08 kg; HOMA‐IR 4.49 ± 0.62 Lean: 3M/3F; mean age 16.0 ± 0.4 years; BMI z‐score 0.05 ± 0.43; fat mass 15.79 ± 2.97 kg; HOMA‐IR 0.61 ± 0.16	Positive for BCAA and IL‐6 levels (*r* = 0.52; *p* = 0.01) and BCAA and log hs‐CRP (*r* = 0.53; *p* = 0.01)
Reddy et al. [74]	MS‐classified adult women	MS with diabetes: 3M/17F; mean age 48 ± 13 years; BMI 30.2 ± 5.6; fat mass N.D.; HOMA‐IR 1.6 (1.1–2.8). MS without diabetes: 7M/22F; mean age 53 ± 9 years; BMI 35.1 ± 6.3; fat mass N.D.; HOMA‐IR 2.8 (1.9–5.8).	From BCAA only isoleucine positive correlated with IL‐6 (*r* = 0.43; *p* = 0.0039)
Katagiri et al. [75]	Healthy adults	482M/526F; mean age 59.5/60.0 ± 5.7 years; BMI 22.2/23.5 ± 2.7/23.5 ± 5.7; fat mass N.D.; HOMA‐IR 0.88/1.04 ± 0.4	No correlation found between BCAA and TNF‐α levels
Lee et al. [76]	Adults with T2D	NFG: 27M/46F; mean age 32 (24–50) years; BMI 21.9 (20.3–24.1); fat mass N.D.; HOMA‐IR 1.34 (0.90–1.80) IFG: 30M/39F; mean age 57 (48–63) years; BMI 24.2 (22.6–25.7); fat mass N.D.; HOMA‐IR 1.82 (1.22–3.26) T2D: 34M/22F; mean age 53 (45–64) years; BMI 24.9 (22.9–28.1); fat mass N.D.; HOMA‐IR 2.74 (2.03–4.27)	Positive for BCAA and IL‐6 levels (*r* = 0.400; *p* = 0.001) and TNF‐α (*r* = 0.464; *p* = 0.001)

Abbreviations: BCAA: branched‐chain amino acid; BMI: body mass index; CPR: c‐reactive protein; IFG: impaired fasting glucose; HOMA‐IR: Homeostatic Model Assessment of Insulin Resistance; MS: metabolic syndrome; NFG: normal fasting glucose; T2D: type 2 diabetes mellitus; IL‐6: interleukin‐6; TNF‐α: tumoral necrose factor alpha.

In adults, the association between BCAA blood levels and inflammatory markers in adult populations appears to vary depending on health status and metabolic conditions (Table 4) [5, 74, 75, 76]. In a study involving adult women classified with metabolic syndrome, Reddy et al. found that among the BCAA, only isoleucine showed a significant positive correlation with IL‐6 levels [74]. Regarding TNF‐α, Katagiri et al. reported no correlation with BCAA in healthy adults [75]. However, Lee et al. identified significant positive correlations between BCAA and both IL‐6 and TNF‐α in T2D patients [76]. This suggests that chronic metabolic disturbances may modulate the inflammatory response to the circulating BCAA levels.

These findings align with growing evidence indicating that chronic low‐grade inflammation plays a key role in various age‐related pathological conditions [77], which presence is marked by elevated proinflammatory cytokines, high glucose and increased BCAA levels may be a contributing factor. Nevertheless, the relationship between BCAA and inflammation is complex, and current knowledge does not support a definitive causal role for these AAs, as most studies suggest that a prior or concurrent inflammatory stimulus is necessary to reveal their involvement in modulating the inflammatory response.

Specific tissue alterations can be related to the pattern of BCAA enzyme catabolism expression seen in murine animals, where a high activity of BCKDH complex is seen in the liver and adipose tissue, with a low activity in skeletal muscle when measured by organ weight as a proportion of total body weight [32, 78]. However, in humans, although the pattern of expression of BCAT mRNA in tissues was similar to that in a murine model, skeletal muscle represented 50%–60% of the BCKDH oxidative capacity [78, 79]. This aspect hampers the development of a direct relation of such results with human studies. Although that, the association presented here between BCAA blood levels and inflammatory markers in adult populations with obesity and/or with diabetes, suggests that chronic metabolic disturbances may modulate the inflammatory response and the circulating BCAA levels. These alterations in pediatric and healthy adult populations were uncommonly seen, the same occurring in wild‐type animals fed an ND [48, 70, 73].

Another point of interest is the potential effects of BCAA supplementation in mice, and this also brings some contradictory results. Gart and colleagues presented an anti‐inflammatory and hypo‐insulinemic valine supplementation potential within a capacity to restore some aspects in a mice model for HFD liver induced‐damage [50]. Interestingly, this was independent of effects on body composition or caloric intake and although the evaluated inflammatory factors in the liver presented a decrease in its expression, no alteration was seen in the protein levels when also evaluated [50]. Notably, a low‐to‐medium isoleucine medium supplementation also emerges as a critical component among BCAAs with potential immunomodulatory capacity in macrophages (increasing NO, IL‐6, and IL‐10), where it influenced the immune cellular responses prior to LPS stimulation, suggesting a priming role that may confer immunomodulatory benefits in early or preclinical stages of inflammation [65]. These do not corroborate with the literature at some point, as studies found that high BCAA levels suppress NO synthesis in macrophages [80, 81, 82], indicating that supra‐physiological doses may also dampen classic inflammatory mediator release. Thus, BCAA effects on immune modulation appear to be dose‐dependent. For instance, while valine or isoleucine supplementation reduced markers of liver steatosis and oxidative stress in some obese or diabetic murine models [50, 68] when Zheng and colleagues evaluated it in lean mice, valine supplementation produced adverse effects [83] This corroborates with a BCAAs beneficial/negative threshold described by Jian and colleagues in avians and confirmed in murine models by Felicianna [66, 67] and agree with the results of an earlier study from Harper and Peters where a protein consumption equilibrium was pursued in the long term by the animals for maintenance of systemic metabolic health [84]. In the Jian and Felicianna studies, they used significantly lower doses than the majority of studies and presented beneficial effects distinctly from higher doses where adverse effects were seen. Interestingly, in another study although BCAA supplementation induced pro‐inflammatory gene expression in visceral adipose tissue under both normal and low protein conditions, it deteriorated insulin intolerance only during the protein restriction condition [85]. This suggests that an AA proportion equilibrium is also necessary to maintain optimum systemic metabolic health and some discrepancy in the assay results can be related to the dose, proportion of each BCAA, and the total AAs content supplemented. Interestingly, in the classical Harper and Peters study they described that distinct from the other AAs, the adaptation of BCAA degradation enzymes in the long term is somehow deficient and BCAA blood levels tend not to accompany the other AA levels [84]. In this context, recent studies on high‐protein diets have also shown important effects on liver function, with long‐term high‐protein intake promoting hepatic triacylglycerol deposition, oxidative stress, and inflammatory signatures in animal models [86], while in human short‐term (6 weeks) dietary interventional studies, isocaloric high‐protein diets reduced intrahepatic lipid content in T2D patients without proportional changes in non‐invasive liver fat indices, highlighting both risk and therapeutic opportunities depending on the experimental conditions and metabolic status [87, 88].

Some of the studies presented here proposed some causality possibilities for the adverse effects of BCAA. Zhao and colleagues demonstrated high BCAA exposure can elevate mitochondrial H₂O₂ levels. Furthermore, after enhancing BCAA catabolism via overexpression of BCKDH, a reduced H₂O₂ formation and consequently lower proinflammatory activation of macrophages were seen. This proves at some point that an impaired BCAA catabolism and a low rate of BCAA or BCKA disappearance can contribute to ROS formation [54]. Also, in another study, a high BCAA concentration activation of mTORC1 leads to the inflammatory transcription factor NF‐κB translocation and an increase in the production of inflammatory factors. These effects were promptly inhibited by the presence of the mTORC1 inhibitor rapamycin in the medium [41]. Together, these results presented that when BCAA alters the oxidative equilibrium and the inflammatory pathway in a cell, a possible adverse outcome will occur.

The complexity of BCAA interactions with inflammation is further emphasized in db/db mice, which exhibit severe metabolic dysfunction, organ damage, and high inflammatory tone. Liu and colleagues observed that high‐BCAA supplementation exacerbated lipid accumulation and macrophage infiltration in db/db mice, while BT2, a BCKDK inhibitor, reversed these effects without significantly affecting systemic metabolic markers. This corroborates the hypothesis that impaired BCAA oxidation contributes to chronic inflammation by promoting macrophage activation, especially in metabolic disease states [51], and points to the importance of a deeper understanding of the physiological response to different doses of BCAA.

Furthermore, the transcriptional regulation of BCAA metabolic pathways by PPAR family members reveals additional layers of complexity. For instance, PPAR‐α and PPAR‐γ overexpression have been associated with increased expression of BCKDH components and reduced circulating BCAA levels [55, 57]. However, again, these effects appear to be tissue‐specific and influenced by genetic and dietary contexts.

## 4 Concluding Remarks

**FIGURE 3:**
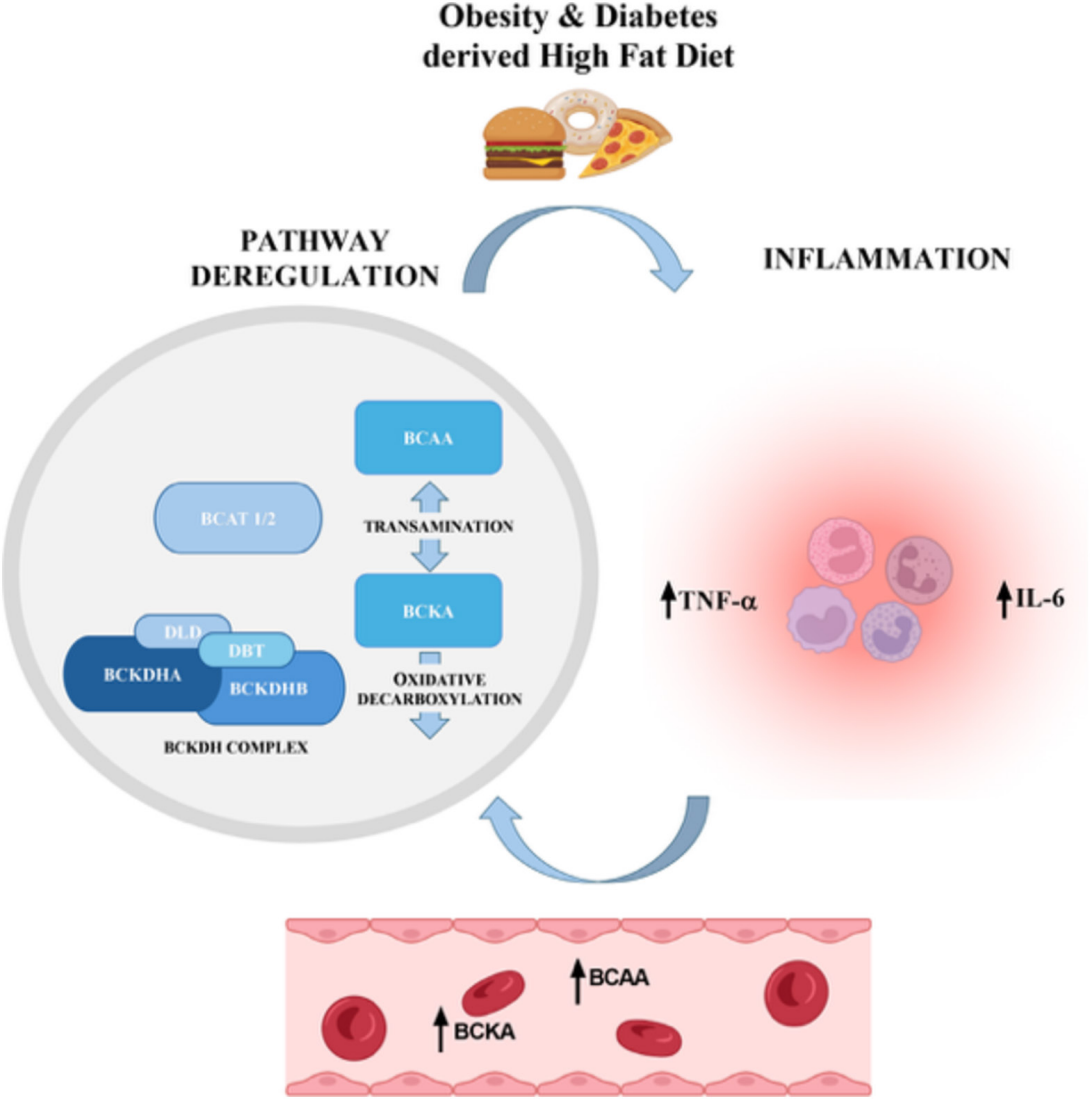
The interplay between BCAA metabolism and inflammation in the obesity and diabetes context. BCAA, branched‐chain amino acid; BCKA, branched‐chain keto acids; BCAT, branched‐chain aminotransferase; BCKDH, branched‐chain ketoacid dehydrogenase; DBT, dihydrolipoamide branched chain transacylase E2; TNF‐α, tumoral necrose factor; IL‐6, interleukin‐6. Created with http://BioRender.com.

Altogether, these findings reinforce the notion that BCAAs exert context and dose‐dependent effects, ranging from beneficial metabolic and anti‐inflammatory responses to deleterious immune activation. The activity of BCKDH and the balance between BCAA and BCKA levels appear to be central regulators of these outcomes (Figure 3). Variations in genetic background, diet, fasting state, and tissue‐specific enzyme expression likely explain the heterogeneity seen across studies. Given the widespread implications of BCAA metabolism in metabolic health, inflammation, and organ integrity, future research should focus on standardized BCAA supplementation protocols and long‐term outcomes across different physiological and pathological states. In particular, understanding the role of individual BCAAs (leucine vs. isoleucine vs. valine), the impact of pharmacological BCAA metabolism modulation (e.g., BT2, PPARs activation), and the interplay between inflammation and BCAA catabolism will be crucial in harnessing the therapeutic potential of BCAA modulation.

## Funding

This work was supported by research grants from Fundação de Apoio à Pesquisa do Estado de Minas Gerais ‐ FAPEMIG, Pro‐Reitoria de Pesquisa (PRPq) UFMG, Conselho Nacional de Desenvolvimento Científico e Tecnológico (CNPq), and Coordenação de Aperfeiçoamento de Pessoal de Nível Superior (CAPES).

## Conflicts of Interest

The authors declare no conflicts of interest.

## Data Availability

The data that support the findings of this study are available from the corresponding author upon reasonable request.

## References

[1] C. E. Kosmas , A. Sourlas , K. Oikonomakis , E. A. Zoumi , A. Papadimitriou , and C. E. Kostara , “Biomarkers of Insulin Sensitivity/Resistance,” Journal of International Medical Research 52, no. 10 (2024): 03000605241285550, 10.1177/03000605241285550.

[2] J. Long , Z. Yang , L. Wang , et al., “Metabolite Biomarkers of Type 2 Diabetes Mellitus and Pre‐Diabetes: A Systematic Review and Meta‐Analysis,” BMC Endocrine Disorders 20, no. 1 (2020): 174, 10.1186/s12902-020-00653-x. 33228610 PMC7685632

[3] X. Zhao , X. Gang , Y. Liu , C. Sun , Q. Han , and G. Wang , “Using Metabolomic Profiles as Biomarkers for Insulin Resistance in Childhood Obesity: A Systematic Review,” Journal Diabetes Research 2016 (2016): 8160545, 10.1155/2016/8160545. PMC4969529 27517054

[4] J. L. Flores‐Guerrero , M. C. J. Osté , L. M. Kieneker , et al., “Plasma Branched‐Chain Amino Acids and Risk of Incident Type 2 Diabetes: Results From the PREVEND Prospective Cohort Study,” Journal of Clinical Medicine 7, no. 12 (2018): 513, 10.3390/jcm7120513. 30518023 PMC6306832

[5] M. A. Connelly , J. Wolak‐Dinsmore , and R. P. F. Dullaart , “Branched Chain Amino Acids Are Associated With Insulin Resistance Independent of Leptin and Adiponectin in Subjects With Varying Degrees of Glucose Tolerance,” Metabolic Syndrome and Related Disorders 15, no. 4 (2017): 183–186, 10.1089/met.2016.0145. 28437198

[6] C. C. Lee , S. M. Watkins , C. Lorenzo , et al., “Branched‐Chain Amino Acids and Insulin Metabolism: The Insulin Resistance Atherosclerosis Study (IRAS),” Diabetes Care 39, no. 4 (2016): 582–588, 10.2337/dc15-2284. 26895884 PMC4806771

[7] T. J. Wang , M. G. Larson , R. S. Vasan , et al., “Metabolite Profiles and the Risk of Developing Diabetes,” Nature Medicine 17, no. 4 (2011): 448–453, 10.1038/nm.2307. PMC3126616 21423183

[8] J. L. Proust , “Recherches Sur Le Principe Qui Assaisonne Les Fromages,” Annales de Chimie Physique 2, no. 10 (1819): 29–49.

[9] H. Braconnot , “Sur La Conversion Des Matieres Animales En Nouvelles Substances Par Le Moyen De L'Acide Sulfurique,” Annales de Chimie Physique 2, no. 13 (1820): 113–125.

[10] Y. Shimomura and R. A. Harris , “Metabolism and Physiological Function of Branched‐Chain Amino Acids: Discussion of Session,” Journal of Nutrition 136 (2006): 232S–233S, 10.1093/jn/136.1.232s. 16365088

[11] G. D. Rose , A. R. Geselowitz , G. J. Lesser , R. H. Lee , and M. H. Zehfus , “Hydrophobicity of Amino Acid Residues in Globular Proteins,” Science (1979) 229, no. 4716 (1985): 834–838, 10.1126/science.4023714. 4023714

[12] P. Burkhard , J. Stetefeld , and S. V. Strelkov , “Coiled Coils: A Highly Versatile Protein Folding Motif,” Trends in Cell Biology 11, no. 2 (2001): 82–88, 10.1016/S0962-8924(00)01898-5. 11166216

[13] Y. Shimomura , T. Murakami , N. Nakai , M. Nagasaki , and R. A. Harris , “3rd Amino Acid Workshop Exercise Promotes BCAA Catabolism: Effects of BCAA Supplementation on Skeletal Muscle during Exercise 1,” (2004), https://academic.oup.com/jn/article/134/6/1583S/4688850. 10.1093/jn/134.6.1583S 15173434

[14] M. Holeček , “Branched‐Chain Amino Acids in Health and Disease: Metabolism, Alterations in Blood Plasma, and as Supplements,” Nutrition & Metabolism 15, no. 1 (2018): 33, 10.1186/s12986-018-0271-1. 29755574 PMC5934885

[15] S. M. Hutson , A. J. Sweatt , and K. F. Lanoue , “4th Amino Acid Assessment Workshop Branched‐Chain Amino Acid Metabolism: Implications for Establishing Safe Intakes,” 1,2. (2005). 10.1093/jn/135.6.1557S 15930469

[16] M. Hargreaves and L. L. Spriet , “Skeletal Muscle Energy Metabolism During Exercise,” Nature Metabolism 2, no. 9 (2020): 817–828, 10.1038/s42255-020-0251-4. 32747792

[17] K. D. Copps and M. F. White , “Regulation of Insulin Sensitivity by Serine/Threonine Phosphorylation of Insulin Receptor Substrate Proteins IRS1 and IRS2,” Diabetologia 55, no. 10 (2012): 2565–2582, 10.1007/s00125-012-2644-8. 22869320 PMC4011499

[18] P. Gumus Balikcioglu , C. Jachthuber Trub , M. Balikcioglu , et al., “Branched‐Chain α‐Keto Acids and Glutamate/Glutamine: Biomarkers of Insulin Resistance in Childhood Obesity,” Endocrinology, Diabetes & Metabolism 6, no. 1 (2023): e388, 10.1002/edm2.388. PMC9836245 36415168

[19] N. Torres , G. López , S. De Santiago , S. M. Hutson , and A. R. Tovar , “Dietary Protein Level Regulates Expression of the Mitochondrial Branched‐Chain Aminotransferase in Rats,” Journal of Nutrition 128, no. 8 (1998): 1368–1375, 10.1093/jn/128.8.1368. 9687558

[20] KHATRA , “Distribution of Branched‐Chain a‐Keto Acid Dehydrogenases in Primate Tissues,” 484. 10.1172/JCI108671 PMC333393 402386

[21] G. Lu , H. Sun , P. She , et al., “Protein Phosphatase 2Cm Is a Critical Regulator of Branched‐Chain Amino Acid Catabolism in Mice and Cultured Cells,” Journal of Clinical Investigation 119, no. 6 (2009): 1678–1687, 10.1172/JCI38151. 19411760 PMC2689111

[22] R. Paxton and R. A. Harris , “Regulation of Branched‐Chain α‐Ketoacid Dehydrogenase Kinase,” Archives of Biochemistry and Biophysics 231, no. 1 (1984): 48–57, 10.1016/0003-9861(84)90361-8. 6721501

[23] S. Polakof , “Acides Aminés à Chaîne Ramifiée et Insulino‐Sensibilité: Amis Ou Ennemis Article Ayant Fait l'objet d'une Conférence Lors Des Journées Francophones de Nutrition De,” (2019).

[24] J. T. Brosnan and M. E. Brosnan , “Branched‐Chain Amino Acids: Enzyme and Substrate Regulation,” Journal of Nutrition 136, no. 1 (2006): 207S–211S, 10.1093/jn/136.1.207S. 16365084

[25] F. G. Banting and C. H. Best , “Nutrition Classics the Internal Secretion of the Pancreas,” (1922). 17582843

[26] J. R. Gavin , J. Roth , D. M. Neville , P. De Meyts , and D. N. Buell , “Insulin‐Dependent Regulation of Insulin Receptor Concentrations: A Direct Demonstration in Cell Culture,” National Academy of Sciences of the United States of America 71, no. 1 (1974): 84–88, 10.1073/pnas.71.1.84. PMC387937 4359334

[27] A. R. Saltiel , “Insulin Resistance in the Defense Against Obesity,” Cell Metabolism 15, no. 6 (2012): 798–804, 10.1016/j.cmet.2012.03.001. 22682220

[28] C. M. Kusminski , P. E. Bickel , and P. E. Scherer , “Targeting Adipose Tissue in the Treatment of Obesity‐Associated Diabetes,” Nature Reviews Drug Discovery 15, no. 9 (2016): 639–660, 10.1038/nrd.2016.75. 27256476

[29] P. J. White , R. W. McGarrah , M. A. Herman , J. R. Bain , S. H. Shah , and C. B. Newgard , “Insulin Action, Type 2 Diabetes, and Branched‐Chain Amino Acids: A Two‐Way Street,” Molecular Metabolism 52 (2021): 101261, 10.1016/j.molmet.2021.101261. 34044180 PMC8513145

[30] M. Roden and G. I. Shulman , “The Integrative Biology of Type 2 Diabetes,” Nature 576, no. 7785 (2019): 51–60, 10.1038/s41586-019-1797-8. 31802013

[31] H. Herrema , J. Lee , Y. Zhou , K. D. Copps , M. F. White , and U. Ozcan , “IRS1Ser307 Phosphorylation Does Not Mediate mTORC1‐Induced Insulin Resistance,” Biochemical and Biophysical Research Communications 443, no. 2 (2014): 689–693, 10.1016/j.bbrc.2013.12.023. 24333417 PMC3926104

[32] J. S. Burrill , E. K. Long , B. Reilly , et al., “Inflammation and ER Stress Regulate Branched‐Chain Amino Acid Uptake and Metabolism in Adipocytes,” Molecular Endocrinology 29, no. 3 (2015): 411–420, 10.1210/me.2014-1275. 25635940 PMC4347289

[33] S. M. Reilly and A. R. Saltiel , “A Complex Role for Adipose Tissue Macrophages,” Nature Reviews. Endocrinology 10, no. 4 (2014): 193–194, 10.1038/nrendo.2014.12. 24492182

[34] A. S. Wedell‐Neergaard , L. Lang Lehrskov , R. H. Christensen , et al., “Exercise‐Induced Changes in Visceral Adipose Tissue Mass Are Regulated by IL‐6 Signaling: A Randomized Controlled Trial,” Cell Metabolism 29, no. 4 (2019): 844–855.e3, 10.1016/j.cmet.2018.12.007. 30595477

[35] M. F. White and C. R. Kahn , “Insulin Action at a Molecular Level—100 Years of Progress,” Molecular Metabolism 52 (2021): 101304, 10.1016/j.molmet.2021.101304. 34274528 PMC8551477

[36] L. Rui , M. Yuan , D. Frantz , S. Shoelson , and M. F. White , “SOCS‐1 and SOCS‐3 Block Insulin Signaling by Ubiquitin‐Mediated Degradation of IRS1 and IRS2,” Journal of Biological Chemistry 277, no. 44 (2002): 42394–42398, 10.1074/jbc.C200444200. 12228220

[37] S. Bonala , S. Lokireddy , C. McFarlane , S. Patnam , M. Sharma , and R. Kambadur , “Myostatin Induces Insulin Resistance via Casitas B‐Lineage Lymphoma b (Cblb)‐Mediated Degradation of Insulin Receptor Substrate 1 (IRS1) in Response to High Calorie Diet Intake,” Journal of Biological Chemistry 291, no. 27 (2016): 14392, 10.1074/jbc.A113.529925. 27371568 PMC4933197

[38] N. J. Hançer , W. Qiu , C. Cherella , Y. Li , K. D. Copps , and M. F. White , “Insulin and Metabolic Stress Stimulate Multisite Serine/Threonine Phosphorylation of Insulin Receptor Substrate 1 and Inhibit Tyrosine Phosphorylation,” Journal of Biological Chemistry 289, no. 18 (2014): 12467–12484, 10.1074/jbc.M114.554162. 24652289 PMC4007441

[39] J. P. De Bandt , X. Coumoul , and R. Barouki , “Branched‐Chain Amino Acids and Insulin Resistance, From Protein Supply to Diet‐Induced Obesity,” Nutrients 15, no. 1 (2023): 68, 10.3390/nu15010068. PMC9824001 36615726

[40] C. J. Lynch and S. H. Adams , “Branched‐Chain Amino Acids in Metabolic Signalling and Insulin Resistance,” Nature Reviews. Endocrinology 10, no. 12 (2014): 723–736, 10.1038/nrendo.2014.171. PMC4424797 25287287

[41] O. Zhenyukh , E. Civantos , M. Ruiz‐Ortega , et al., “High Concentration of Branched‐Chain Amino Acids Promotes Oxidative Stress, Inflammation and Migration of Human Peripheral Blood Mononuclear Cells via mTORC1 Activation,” Free Radical Biology & Medicine 104 (2017): 165–177, 10.1016/j.freeradbiomed.2017.01.009. 28089725

[42] Y. Dong , X. Zhang , R. Miao , et al., “Branched‐Chain Amino Acids Promotes the Repair of Exercise‐Induced Muscle Damage via Enhancing Macrophage Polarization,” Frontiers in Physiology 13 (2022): 1037090, 10.3389/fphys.2022.1037090. 36561213 PMC9763461

[43] H. Yao , K. Li , J. Wei , Y. Lin , and Y. Liu , “The Contradictory Role of Branched‐Chain Amino Acids in Lifespan and Insulin Resistance,” Frontiers in Nutrition 10 (2023): 1189982, 10.3389/fnut.2023.1189982. 37408986 PMC10318341

[44] T. U. Maioli , J. L. Gonçalves , M. C. G. Miranda , et al., “High Sugar and Butter (HSB) Diet Induces Obesity and Metabolic Syndrome With Decrease in Regulatory T Cells in Adipose Tissue of Mice,” Inflammation Research 65, no. 2 (2016): 169–178, 10.1007/s00011-015-0902-1. 26650032

[45] K. A. Lo , A. Labadorf , N. J. Kennedy , et al., “Analysis of In Vitro Insulin‐Resistance Models and Their Physiological Relevance to In Vivo Diet‐Induced Adipose Insulin Resistance,” Cell Reports 5, no. 1 (2013): 259–270, 10.1016/j.celrep.2013.08.039. 24095730 PMC3874466

[46] R. Liu , H. Li , W. Fan , et al., “Leucine Supplementation Differently Modulates Branched‐Chain Amino Acid Catabolism, Mitochondrial Function and Metabolic Profiles at the Different Stage of Insulin Resistance in Rats on High‐Fat Diet,” Nutrients 9, no. 6 (2017): 565, 10.3390/nu9060565. 28574481 PMC5490544

[47] B. H. Choi , S. Hyun , and S. H. Koo , “The Role of BCAA Metabolism in Metabolic Health and Disease,” Experimental & Molecular Medicine 56, no. 7 (2024): 1552–1559, 10.1038/s12276-024-01263-6. 38956299 PMC11297153

[48] F. Zhang , S. Zhao , W. Yan , et al., “Branched Chain Amino Acids Cause Liver Injury in Obese/Diabetic Mice by Promoting Adipocyte Lipolysis and Inhibiting Hepatic Autophagy,” eBioMedicine 13 (2016): 157–167, 10.1016/j.ebiom.2016.10.013. 27843095 PMC5264279

[49] K. Lian , C. Du , Y. Liu , et al., “Impaired Adiponectin Signaling Contributes to Disturbed Catabolism of Branched‐Chain Amino Acids in Diabetic Mice,” Diabetes 64, no. 1 (2015): 49–59, 10.2337/db14-0312. 25071024

[50] E. Gart , W. van Duyvenvoorde , M. P. M. Caspers , et al., “Intervention With Isoleucine or Valine Corrects Hyperinsulinemia and Reduces Intrahepatic Diacylglycerols, Liver Steatosis, and Inflammation in Ldlr−/−.Leiden Mice With Manifest Obesity‐Associated NASH,” FASEB Journal 36, no. 8 (2022): e22435, 10.1096/fj.202200111R. 35830259 PMC12166278

[51] S. Liu , L. Li , P. Lou , et al., “Elevated Branched‐Chain α‐Keto Acids Exacerbate Macrophage Oxidative Stress and Chronic Inflammatory Damage in Type 2 Diabetes Mellitus,” Free Radical Biology & Medicine 175 (2021): 141–154, 10.1016/j.freeradbiomed.2021.08.240. 34474107

[52] L. M. Li , X. Liu , L. Wang , et al., “A Novel Dual Eigen‐Analysis of Mouse Multi‐Tissues' Expression Profiles Unveils New Perspectives Into Type 2 Diabetes,” Scientific Reports 7, no. 1 (2017): 5044, 10.1038/s41598-017-05405-x. 28698587 PMC5506042

[53] H. Huang , H. Chen , Y. Yao , and X. Lou , “Branched‐Chain Amino Acids Supplementation Induces Insulin Resistance and Pro‐Inflammatory Macrophage Polarization via INFGR1/JAK1/STAT1 Signal Pathway,” Molecular Medicine 30, no. 1 (2024): 149, 10.1186/s10020-024-00894-9. 39267003 PMC11391606

[54] S. Zhao , L. Zhou , Q. Wang , et al., “Elevated Branched‐Chain Amino Acid Promotes Atherosclerosis Progression by Enhancing Mitochondrial‐to‐Nuclear H_2_O_2_‐Disulfide HMGB1 in Macrophages,” Redox Biology 62 (2023): 102696, 10.1016/j.redox.2023.102696. 37058999 PMC10130699

[55] H. Takahashi , K. Sanada , H. Nagai , et al., “Over‐Expression of PPARα in Obese Mice Adipose Tissue Improves Insulin Sensitivity,” Biochemical and Biophysical Research Communications 493, no. 1 (2017): 108–114, 10.1016/j.bbrc.2017.09.067. 28919422

[56] Y. Wang , N. Zhao , Y. Meng , et al., “Bcat2‐Mediated Branched‐Chain Amino Acid Catabolism Is Linked to the Aggravated Inflammation in Obese With Psoriasis Mice,” Molecular Nutrition & Food Research 68, no. 8 (2024): e2300720, 10.1002/mnfr.202300720. 38581348

[57] P. G. Blanchard , R. J. Moreira , É. Castro , et al., “PPARγ Is a Major Regulator of Branched‐Chain Amino Acid Blood Levels and Catabolism in White and Brown Adipose Tissues,” Metabolism 89 (2018): 27–38, 10.1016/j.metabol.2018.09.007. 30316815

[58] Q. Gong , R. Zhang , F. Wei , et al., “SGLT2 Inhibitor‐Empagliflozin Treatment Ameliorates Diabetic Retinopathy Manifestations and Exerts Protective Effects Associated With Augmenting Branched Chain Amino Acids Catabolism and Transportation in db/db Mice,” Biomedicine & Pharmacotherapy 152 (2022): 113222, 10.1016/j.biopha.2022.113222. 35671581

[59] J. M. Vargas‐Morales , R. Guizar‐Heredia , A. L. Méndez‐García , et al., “Association of BCAT2 and BCKDH Polymorphisms With Clinical, Anthropometric and Biochemical Parameters in Young Adults,” Nutrition, Metabolism, and Cardiovascular Diseases 31, no. 11 (2021): 3210–3218, 10.1016/j.numecd.2021.07.011. 34511290

[60] H. K. Pedersen , V. Gudmundsdottir , H. B. Nielsen , et al., “Human Gut Microbes Impact Host Serum Metabolome and Insulin Sensitivity,” Nature 535, no. 7612 (2016): 376–381, 10.1038/nature18646. 27409811

[61] M. A. Herman , P. She , O. D. Peroni , C. J. Lynch , and B. B. Kahn , “Adipose Tissue Branched Chain Amino Acid (BCAA) Metabolism Modulates Circulating BCAA Levels,” Journal of Biological Chemistry 285, no. 15 (2010): 11348–11356, 10.1074/jbc.M109.075184. 20093359 PMC2857013

[62] S. M. Hutson , D. Fenstermacher , and C. Mahar , “Role of Mitochondrial Transamination in Branched Chain Amino Acid Metabolism,” Journal of Biological Chemistry 263, no. 8 (1988): 3618–3625, 10.1016/s0021-9258(18)68969-0. 3346211

[63] K. Peyrollier , E. Hajduch , A. S. Blair , R. Hyde , and H. S. Hundal , “L‐Leucine Availability Regulates Phosphatidylinositol 3‐Kinase, P70 S6 Kinase and Glycogen Synthase Kinase‐3 Activity in L6 Muscle Cells: Evidence for the Involvement of the Mammalian Target of Rapamycin (MTOR) Pathway in the L‐Leucine‐Induced Up‐Regulation of System A Amino Acid Transport,” Biochemical Journal 350 (2000): 361–368. 10947949 PMC1221262

[64] S. Khatua , J. Simal‐Gandara , and K. Acharya , “Understanding Immune‐Modulatory Efficacy In Vitro,” Chemico‐Biological Interactions 352 (2022): 109776, 10.1016/j.cbi.2021.109776. 34906553 PMC8665649

[65] A. Bonvini , M. M. Rogero , A. Y. Coqueiro , et al., “Effects of Different Branched‐Chain Amino Acids Supplementation Protocols on the Inflammatory Response of LPS‐Stimulated RAW 264.7 Macrophages,” Amino Acids 53, no. 4 (2021): 597–607, 10.1007/s00726-021-02940-w. 33715068

[66] E. K. Lo , C. Chen , M. J. Ismaiah , F. Zhang , H. K. Leung , and H. El‐Nezami , “Low‐Dose Valine Attenuates Diet‐Induced Metabolic Dysfunction‐Associated Steatotic Liver Disease (MASLD) in Mice by Enhancing Leptin Sensitivity and Modulating the Gut Microbiome,” Molecular Metabolism 90 (2024): 102059, 10.1016/j.molmet.2024.102059. 39489290 PMC11616088

[67] H. Jian , S. Miao , Y. Liu , et al., “Dietary Valine Ameliorated Gut Health and Accelerated the Development of Nonalcoholic Fatty Liver Disease of Laying Hens,” Oxidative Medicine and Cellular Longevity 2021, no. 1 (2021): 4704771, 10.1155/2021/4704771. 34484560 PMC8410442

[68] D. Terakura , M. Shimizu , J. Iwasa , et al., “Preventive Effects of Branched‐Chain Amino Acid Supplementation on the Spontaneous Development of Hepatic Preneoplastic Lesions in C57BL/KsJ‐db/db Obese Mice,” Carcinogenesis 33, no. 12 (2012): 2499–2506, 10.1093/carcin/bgs303. 23027617

[69] G. S. dos Feijó , J. Jantsch , L. L. Correia , et al., “Neuroinflammatory Responses Following Zinc or Branched‐Chain Amino Acids Supplementation in Obese Rats,” Metabolic Brain Disease 37, no. 6 (2022): 1875–1886, 10.1007/s11011-022-00996-5. 35556196

[70] W. Perng , S. L. Rifas‐Shiman , S. McCulloch , et al., “Associations of Cord Blood Metabolites With Perinatal Characteristics, Newborn Anthropometry, and Cord Blood Hormones in Project Viva,” Metabolism 76 (2017): 11–22, 10.1016/j.metabol.2017.07.001. 28987236 PMC5675164

[71] W. Perng , M. W. Gillman , A. F. Fleisch , et al., “Metabolomic Profiles and Childhood Obesity,” Obesity 22, no. 12 (2014): 2570–2578, 10.1002/oby.20901. 25251340 PMC4236243

[72] R. G. Cosentino , J. R. Churilla , S. Josephson , et al., “Branched‐Chain Amino Acids and Relationship With Inflammation in Youth With Obesity: A Randomized Controlled Intervention Study,” Journal of Clinical Endocrinology and Metabolism 106, no. 11 (2021): 3129–3139, 10.1210/clinem/dgab538. 34286837

[73] J. Bugajska , J. Berska , M. Wójcik , and K. Sztefko , “Amino Acid Profile in Overweight and Obese Prepubertal Children—Can Simple Biochemical Tests Help in the Early Prevention of Associated Comorbidities?,” Frontiers in Endocrinology 14 (2023): 1274011, 10.3389/fendo.2023.1274011. 37964971 PMC10641253

[74] P. Reddy , J. Leong , and I. Jialal , “Amino Acid Levels in Nascent Metabolic Syndrome: A Contributor to the Pro‐Inflammatory Burden,” Journal of Diabetes and its Complications 32, no. 5 (2018): 465–469, 10.1016/j.jdiacomp.2018.02.005. 29559272

[75] R. Katagiri , A. Goto , S. Budhathoki , et al., “Association Between Plasma Concentrations of Branched‐Chain Amino Acids and Adipokines in Japanese Adults Without Diabetes,” Scientific Reports 8, no. 1 (2018): 1043, 10.1038/s41598-018-19388-w. 29348480 PMC5773488

[76] S. G. Lee , Y. S. Yim , Y. Lee , et al., “Fasting Serum Amino Acids Concentration Is Associated With Insulin Resistance and Pro‐Inflammatory Cytokines,” Diabetes Research and Clinical Practice 140 (2018): 107–117, 10.1016/j.diabres.2018.03.028. 29601913

[77] D. Furman , J. Campisi , E. Verdin , et al., “Chronic Inflammation in the Etiology of Disease Across the Life Span,” Nature Medicine 25, no. 12 (2019): 1822–1832, 10.1038/s41591-019-0675-0. PMC7147972 31806905

[78] SURYAWAN , “A Molecular Model of Human Branched‐Chain Amino Acid Metabolism,” (1998). 10.1093/ajcn/68.1.72 9665099

[79] M. D. Neinast , C. Jang , S. Hui , et al., “Quantitative Analysis of the Whole‐Body Metabolic Fate of Branched‐Chain Amino Acids,” Cell Metabolism 29, no. 2 (2019): 417–429, 10.1016/j.cmet.2018.10.013. 30449684 PMC6365191

[80] J. Bryk , J. B. Ochoa , M. I. T. Correia , V. Munera‐Seeley , and P. J. Popovic , “Effect of Citrulline and Glutamine on Nitric Oxide Production in RAW 264.7 Cells in an Arginine‐Depleted Environment,” Journal of Parenteral and Enteral Nutrition 32, no. 4 (2008): 377–383, 10.1177/0148607108319807. 18596308

[81] J. H. Lee , E. Park , H. J. Jin , et al., “Anti‐Inflammatory and Anti‐Genotoxic Activity of Branched Chain Amino Acids (BCAA) in Lipopolysaccharide (LPS) Stimulated RAW 264.7 Macrophages,” Food Science and Biotechnology 26, no. 5 (2017): 1371–1377, 10.1007/s10068-017-0165-4. 30263672 PMC6049802

[82] R. De Simone , F. Vissicchio , C. Mingarelli , et al., “Branched‐Chain Amino Acids Influence the Immune Properties of Microglial Cells and Their Responsiveness to Pro‐Inflammatory Signals,” Biochimica et Biophysica Acta (BBA) ‐ Molecular Basis of Disease 1832, no. 5 (2013): 650–659, 10.1016/j.bbadis.2013.02.001. 23402925

[83] H. Y. Zheng , L. Wang , R. Zhang , R. Ding , C. X. Yang , and Z. Q. Du , “Valine Induces Inflammation and Enhanced Adipogenesis in Lean Mice by Multi‐Omics Analysis,” Frontiers in Nutrition 11 (2024): 1379390, 10.3389/fnut.2024.1379390. 38803448 PMC11128663

[84] A. E. Harper and J. C. Peters , “Protein Intake, Brain Amino Acid and Serotonin Concentrations and Protein Self‐Selection,” Journal of Nutrition 119, no. 5 (1989): 677–689. 2656935 10.1093/jn/119.5.677

[85] W. C. Mu , E. Vanhoosier , C. M. Elks , and R. W. Grant , “Long‐Term Effects of Dietary Protein and Branched‐Chain Amino Acids on Metabolism and Inflammation in Mice,” Nutrients 10, no. 7 (2018): 918, 10.3390/nu10070918. 30021962 PMC6073443

[86] R. Díaz‐Rúa , J. Keijer , A. Palou , E. M. van Schothorst , and P. Oliver , “Long‐Term Intake of a High‐Protein Diet Increases Liver Triacylglycerol Deposition Pathways and Hepatic Signs of Injury in Rats,” Journal of Nutritional Biochemistry 46 (2017): 39–48, 10.1016/j.jnutbio.2017.04.008. 28454041

[87] M. Markova , O. Pivovarova , S. Hornemann , et al., “Isocaloric Diets High in Animal or Plant Protein Reduce Liver Fat and Inflammation in Individuals With Type 2 Diabetes,” Gastroenterology 152, no. 3 (2017): 571–585.e8, 10.1053/j.gastro.2016.10.007. 27765690

[88] S. Kabisch , M. Markova , S. Hornemann , et al., “Liver Fat Scores Do Not Reflect Interventional Changes in Liver Fat Content Induced by High‐Protein Diets,” Scientific Reports 11, no. 1 (2021): 8843, 10.1038/s41598-021-87360-2. 33893355 PMC8065150

